# 

**Published:** 1879-10

**Authors:** 


*Vol. XXXIII.* **OCTOBER, 1879.** *No. 10.*

THE

### DENTAL REGISTER,

A Monthly Journal Devoted to the Interests of the Profession.

EDITED BY J. TAFT, D. D. S.,

AND

PUBLISHED BY SPENCER & CROCKER,

OHIO DENTAL DEPOT,

Nos. 117, 119, 121 West Fifth St., Cincinnati, O.

TERMS: $2.50 Per Annum, in Advance; Single Copies 25c.

